# Predicting the dispersal and invasion dynamics of ambrosia beetles through demographic reconstruction and process-explicit modeling

**DOI:** 10.1038/s41598-024-57590-1

**Published:** 2024-03-30

**Authors:** Lucas A. Fadda, Luis Osorio-Olvera, Luis A. Ibarra-Juárez, Jorge Soberón, Andrés Lira-Noriega

**Affiliations:** 1 Red de Estudios Moleculares Avanzados, Instituto de Ecología A.C., Carretera antigua a Coatepec 351, El Haya, C. P. 91073 Xalapa, Veracruz Mexico; 2 Laboratorio de Ecoinformática de la Biodiversidad, Departamento de Ecología de la Biodiversidad, Instituto de Ecología, Universidad Nacional Autónoma de México, Mexico City, México; 3 Biodiversity Institute, University of Kansas, Lawrence, KS 66045 USA; 4 Laboratorio Nacional Conahcyt de Biología del Cambio Climático, CONAHCyT, Ciudad de México, México; 5 Instituto de Ecología A.C., Red de Estudios Moleculares Avanzados, Carretera Antigua a Coatepec 351, El Haya, Xalapa, Veracruz México

**Keywords:** Ecology, Biogeography

## Abstract

Evaluating potential routes of invasion of pathogens and vectors of sanitary importance is essential for planning and decision-making at multiple scales. An effective tool are process-explicit models that allow coupling environmental, demographic and dispersal information to evaluate population growth and range dynamics as a function of the abiotic conditions in a region. In this work we simulate multiple dispersal/invasion routes in Mexico that could be taken by ambrosia beetles and a specific symbiont, *Harringtonia lauricola*, responsible for a severe epiphytic of Lauraceae in North America. We used *Xyleborus bispinatus* Eichhoff 1868 as a study subject and estimated its demography in the laboratory in a temperature gradient (17, 20, 26, 29, 35 °C), which we then used to parameterize a process-based model to estimate its metapopulation dynamics. The maximum intrinsic growth rate of *X. bispinatus* is 0.13 with a thermal optimum of 26.2 °C. The models suggest important regions for the establishment and dispersal the states of Veracruz, Chiapas and Oaxaca (high host and secondary vectors diversity), the Isthmus of Tehuantepec (connectivity region), and Michoacán and Jalisco (important avocado plantations). The use of hybrid process-based models is a promising tool to refine the predictions applied to the study of biological invasions and species distributions.

## Introduction

Understanding the processes and mechanisms that determine biological invasions have captivated ecologists and biogeographers for decades^1–4^. The dynamics of species’ distributions have been modelled mostly using partial differential equations (or their discrete counterparts) with coefficients constant in time and space^5–10^. However, in any real application, the equations are not autonomous and its coefficients are not constant. These coefficients can in part be estimated as functions of the ecological niche of the species^11^, which to a larger extent, determines the demographic parameters and metapopulation dynamics that eventually become apparent in the abundance and distribution of species^12–16^. Specifically, there is a close relationship between the position of the populations of a species in the ecological niche space and its intrinsic growth rate^17^, which increases towards the center of the fundamental niche^18,19^.

The study of the relationship of a species’ position in its ecological niche and their demographic parameters has been explored using several ecological niche modeling algorithms^20–24^, resulting in multiple applications of biological importance^23^. However, these approximations are generally made from correlative approaches^3,25^ (i.e., static models) that lack explicit population processes such as birth, mortality and migration^26–28^, thus making it difficult to understand the mechanisms that determine the patterns of distribution and abundance of species^29–31^. An alternative to understand the relationship between population processes and the ecological niche comes from including estimates of demographic parameters through life history response experiments^32^ under controlled laboratory conditions^33–36^, that provide empirical evidence that population growth and survival often have a Gaussian response to environmental gradients^24,37–39^. Population growth is probably one of the most important variables to consider when it comes to demographic estimates, since it unifies and links the various facets of population ecology, allowing for the estimation of the point of maximum suitability within its ecological niche (i.e., optimal conditions), and thus understanding how the influence of environmental stress affects species^32^. This can be mathematically represented by an *n*-dimensional ellipsoid^40,^ similar to Maguire’s proposal^41^, which explores the relationship between the structure of the ecological niche and the performance of the species, in strict adherence to Hutchinson's original definition of the fundamental niche^11^.

However, correlative ecological niche models are based on the equilibrium assumption that occupancy has reached a steady state with respect to relevant environmental gradients^42,43^ and, while predictive and useful, the parameters of such statistical models can seldom be interpreted as lower-level processes, an important oversimplification^44^. This is even more problematic in cases of biological invasions when modeling the distribution of a species of economic importance and inferences about the dynamic process of propagation are required^43,45,46^. Process-explicit models make it possible to relax the steady-state assumption of correlative models^47^ by dynamically integrating environmental and demographic information^48^. Including parameters that fit the data and allowing biotic interactions makes it possible to obtain simulations capable of reproducing multiple abundance and dispersal patterns at macroecological scales^49–51^ in diverse spatial and temporal contexts^16,26,48,52,53^. As a result, these models enable the detection of causal implications regarding specific mechanisms for reproducing distributional patterns and identifying non-obvious behaviors based on ecological and biogeographic hypotheses^54^. This is achievable because they offer the flexibility to modify parameters related to demography, dispersal, and environment^43,55–59^.

In practical applications, such as agriculture and forestry, these modeling algorithms prove instrumental in predicting the potential distribution of invasive pests^53,60–65^. Bark and ambrosia beetles, responsible for diverse epiphytes in ecosystems around the world^66,67^ are a notable example of how this tools could help to predict population outbreaks^68^ and assessing their distribution under the influence of climate change^61,69–71^. These reliable preliminary hypotheses about the environmental requirements and suitable sites for potential invasions^72^, aid decision making and mitigate the difficulties and challenges associated with their management^73,74^.

In the United States, the introduction of the Asian ambrosia beetles *Xyleborus glabratus* Eichhoff and *Euwallacea* spp. and its symbiont fungi were responsible for the death of countless trees in the country’s native and cultivated ecosystems due to the exotic phytopathogenic agents they carry^75–78^. Their accelerated dispersal has been largely due to anthropogenic factors, like presence in containers, to the movement of timber across large geographic extents^79–81^. The above, combined with the capacity of the beetles to reproduce asexually (haplodiploidy), have positioned them amongst the most successful colonizers in the world^67,82^. Since the presence of *X. glabratus* was recorded in the United States in 2002^80,81^, an increased mortality of red bay (*Persea borbonia*) was reported, from 10% to more than 90% in a period of 15 months in South Carolina and Florida. These mortality rates were largely related to the presence of *Xyleborus*’ symbiont, the fungus *Harringtonia lauricola*^83^ (formerly *Raffaelea lauricola*^77,78^). In addition, it is estimated that in Florida the losses due to decreased yield and increased production costs caused by this disease in avocado (*Persea americana* Mill.) can range from 183 to 356 million dollars^84^. In a similar way, in California, the complex *Euwallacea* spp., which includes three genetically different but morphologically indistinguishable species^85^, have caused the death, in just three years, of 120,000 willows (*Salix* spp.) by their symbionts of the genus *Fusarium* sp*.* This represents approximately 30% of the native trees of this state^86,87^. However, the most distinctive feature of the *Euwallacea* spp. is its wide polyphagy. According to the study carried out by Eskalen et al*.*^75^ of the 335 tree species observed, 207 representing 58 plant families, showed signs and symptoms compatible with *Euwallacea* spp. attacks. Additionally, they reported that 19 tree species could serve as reproductive hosts in North America, many of which include forest trees, landscape trees, and avocado plantations. This represents a threat to a large diversity of ecosystems^75,88,89^. Mexico is the country with the largest avocado production in the world. In 2020, avocado activity supported around 310,000 direct jobs and 78,000 indirect jobs in the state of Michoacán alone. A large number of Mexican families depend on this crop (https://www.gob.mx/senasica/articulos/aguacate-michoacano-igual-a-empleo-y-bienestar).

*Euwallacea* spp. have been reported in Tijuana^90^ and *X. glabratus* in Texas^91,92^. This in combination with the difficulty to detect the beetles in containers or cargo created a phytosanitary alert^93^. In addition to the direct impacts to the avocado industry, Mexico is a diversity center for Lauraceae and other hosts (known or potential) of phytopathogenic fungi. The states of Chiapas, Oaxaca and Veracruz accumulate 90% of the species for this plant family countrywide (120 species)^63,94^. Moreover, Veracruz has 14 of the 18 native *Xyleborus* species reported for Mexico^95,96^. An invasion of Lauraceae’s phytopathogens spread by *Xyleborus*^97,98^ would represent an ecological disaster to Mexico. In view of the above, it is indispensable for Mexico to understand the potential for dispersal and establishment of ambrosia beetles in the country.

Such a problem can be explored by simulations based on process-explicit models that combine aspects of a species’ fundamental niche with its dispersal capabilities. Because of high restrictions to work with species under phytosanitary restrictions, the task of manipulating them to acquire relevant biological information necessary for parameterizing models may be impossible. This is typical of species that have a quarantine status, as is the current situation in Mexico^99^. An alternative is to make inferences about different traits and behavior through the use of species that are morphologically, phylogenetically and functionally similar^100–102^, which also coincides with them having similar environmental requirements^100,101,103,104^. However, parameterizing models for species subject to phytosanitary restrictions can pose significant challenges. Obtaining fundamental biological information necessary for this task often relies on studies conducted under controlled conditions, such as laboratory settings, greenhouses, or specific field conditions, which can be difficult to access^53^. According to Briscoe et al*.*^48^, two key barriers restrict the use of process-explicit models: data availability and accessibility of methods. In the first case, although there are databases available to obtain information on species, they are often incomplete (e.g., COMPADRE [https://compadre-db.org/Data] only includes two species of Curculionidae, *S. ventralis* and *H. hampei*) or are not entirely freely accessible. Regarding the accessibility of information where software does exist^105^, it may only support a subset of methods, and a lack of technical knowledge can impede their use, thus limiting potential applications. However, there is optimism due to the increase in data collection in databases in recent years. Coupled with the availability of freely accessible environmental layers at fine resolutions, current computing power, and growing knowledge applied to software development, these factors are expected to contribute significantly to overcoming the aforementioned shortcomings.

Although explicit examples that leverage morphological, phylogenetic, or environmental information among species to categorize them as analogous are few, there is implicit evidence in many works indicating the closeness they share in several of these aspects. An instance of this phenomenon is observed with ambrosia beetles. In recent years, it has been confirmed that the sister species *Xyleborus affinis* Eichhoff 1868, *X. volvulus* (F.) 1775, and *X. bispinatus* Eichhoff 1868 (until 2006 not differentiated from *X. ferrugineus*^106^) can function as effective secondary vectors of *H. lauricola*^79,98,107–112^. Among the three species of ambrosial beetles, *X. bispinatus* stands out due to its extensive geographic distribution across the American continent and recent occurrence in Europe and New Guinea^113^, its ability to survive in a diverse range of hosts (including avocado)^107^, and its association with the aforementioned phytopathogenic fungus^114^. Furthermore, considering its phylogenetic proximity^102^ and environmental affinity^25,115^ with *X. glabratus*, we propose that this species could serve as a model for estimating the demography of *X. glabratus* and suggesting management plans, particularly given the phytosanitary constraints associated with working with this exotic species.

Eradication of an invasive species over large areas is extremely difficult when it has been established for a long time^43,116,117^. The alternative approach is to predict and understand invasion pathways and processes in order to prioritize strategies to control its arrival and spread^118,119^. In this study, we use an explicit process-based dynamic model to estimate the processes and resulting geographic patterns of ambrosia beetles in Mexico. We parameterize the model using demographic rates obtained from laboratory rearing for *X. bispinatus* and niche modeling. We demonstrate how simulations from process-based models can complement phytosanitary tools by considering environmental, demographic, and dispersal aspects. This approach proves valuable for suggesting monitoring and early detection protocols to contribute to decision-making in programs for the prevention, management, and eradication of invasive species.

## Results

### Laboratory

#### Adjustment of the growth function and optimal rate of growth

**Figure 1:**
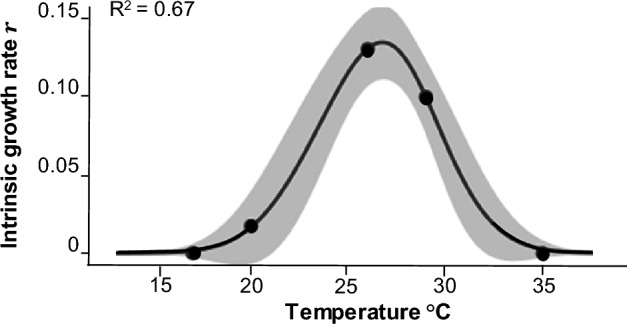
Mathematical function of growth rate of *X. bispinatus* at different temperatures considering a 95% confidence interval.

Survivorship curves and oviposition rates of *X. bispinatus* were obtained to estimate the intrinsic growth rate ${\varvec{r}}$ as function of temperature and the optimum temperature $\left({{\varvec{t}}}_{{\varvec{o}}{\varvec{p}}{\varvec{t}}}\right)$ at which the maximum growth rate $\left({{\varvec{r}}}_{{\varvec{m}}{\varvec{a}}{\varvec{x}}}\right)$ occurs, and kurtosis of the fitted curve $\left({{\varvec{v}}}_{0}\right)$ which we interpreted as a measure of niche breadth. ${{\varvec{r}}}_{{\varvec{m}}{\varvec{a}}{\varvec{x}}}$ occurs at 26.26 °C $\left({{\varvec{t}}}_{{\varvec{o}}{\varvec{p}}{\varvec{t}}}\right)$. ${{\varvec{r}}}_{{\varvec{m}}{\varvec{a}}{\varvec{x}}}$ = 0.13 individuals/individuals/day (Table 1). The coefficient of determination of the curve was R^2^ = 0.67, while the hypothesis tests for the non-linear regression parameters showed a high level of significance (Fig. 1; Table 1).

**Table 1:** Parameters of the mathematical growth rate function of *X. bispinatus* as a function of temperature.

Parameters	Estimate	Standard error	t value	Pr ( >|t|)
*rmax*	0.1314	0.0113	11.6800	< 0.0001***
*V*0	0.0521	0.0160	3.2600	< 0.001**
*topt*	26.2624	0.4137	63.4800	< 0.0001***

Statistical significance: 0 ‘***’, 0.001 ‘**’, 0.01 ‘*’, 0.05 ‘’, residual standard error 0.03771, 47 degrees of freedom.

### Dispersal dynamics modeling

#### Environmental suitability and correlative model using the intrinsic growth rate *r*

**Figure 2:**
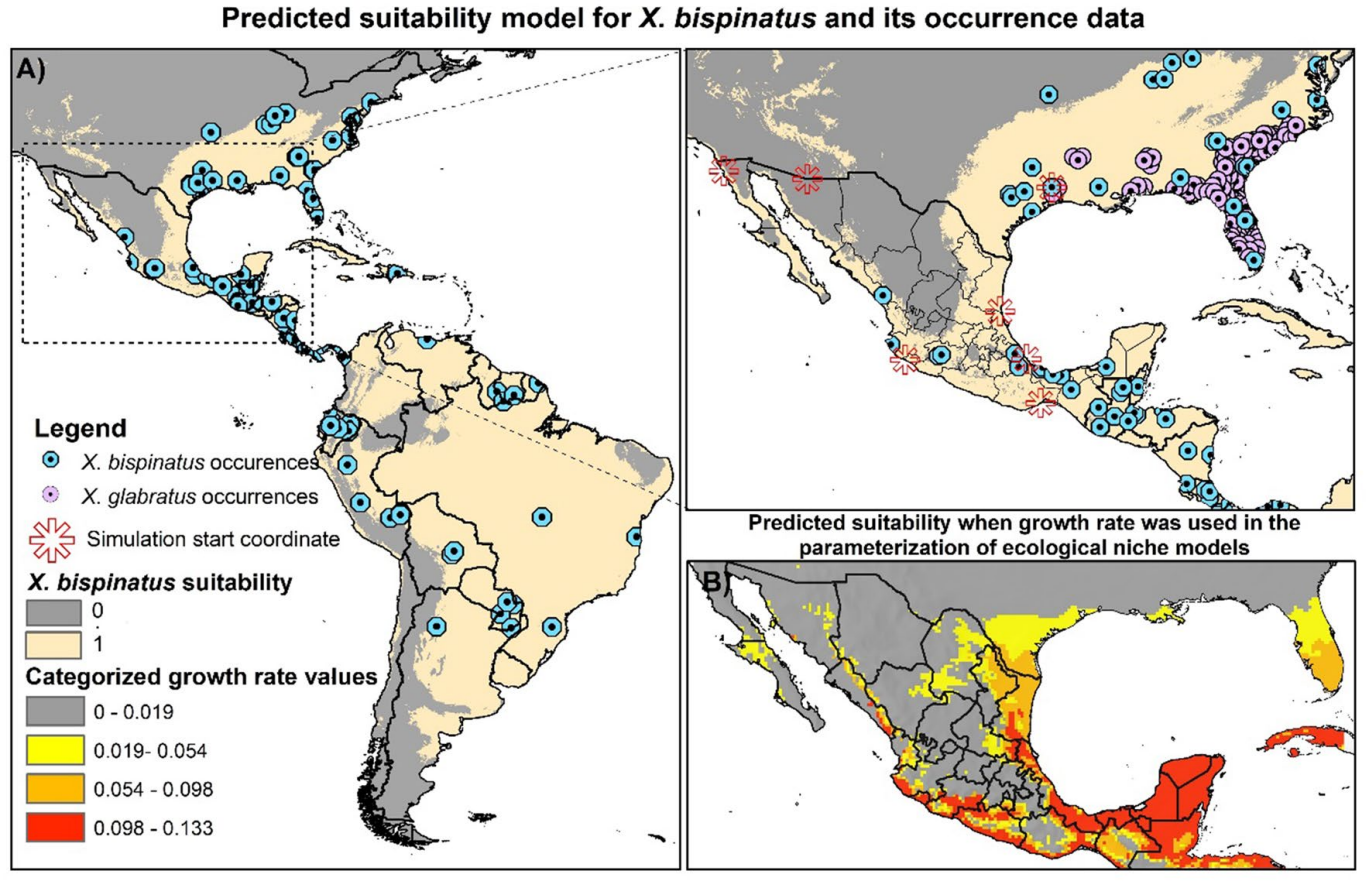
(**A**) Map with the suitable sites estimated from the ecological niche modeling, the records of *X. bispinatus* presence, and the starting coordinates used for the spatiotemporal simulations. (**B**) Projection in geography of *X. bispinatus* growth estimated from the intrinsic growth rate function (*r*_*i*_ map) used for the construction of the process-explicit models. This figure was generated in ArcGIS ver. 10.4 (https://www.arcgis.com/features/index.html).

The selected correlative model incorporated environmental layers bio4, bio9, bio10, bio12, bio15, bio17, and bio18^120^ and satisfied the evaluation criteria based on the following parameters: omission rate in test data of 0.074, omission rate in training data of 0.063, average omission rate of training and test data of 0.068, background prevalence of 0.816, and passed the binomial test (*P*-value = 0.029). This model showed sites with environmental suitability for the species along the slope of the Gulf of Mexico and in some states on the Pacific coast (Fig. 2A). The *r*_*i*_ map (Fig. 2B) showed areas with different levels of suitability in the states of Veracruz, Oaxaca, Tabasco, and Chiapas, especially important due to the occurrence of higher numbers of species of Lauraceae hosts and secondary vectors, as well as in the Isthmus of Tehuantepec, which also connects the western and eastern regions of the country. Highly suitable climates were also recorded in the Yucatan peninsula and the avocado-producing area comprised of Michoacán, Jalisco, Nayarit, part of the State of Mexico, Morelos, and Colima. Towards the north of Mexico, a decrease in *r*_*i*_ values were observed, which could mean a natural barrier for the dispersal and establishment of the species at these latitudes (Fig. 2B).

#### Metapopulation process-explicit model

**Figure 3:**
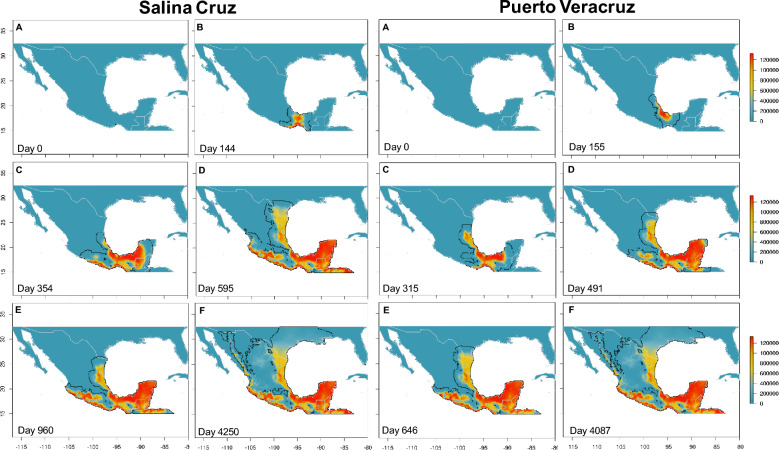
Predicted invasion dynamics and abundance of the ambrosial complex *X. bispinatus*–*H. lauricola* from the port of Salina Cruz and Veracruz at different time periods. Maps were generated with the raster package ver. 3.6 and wesanderson color palette (https://github.com/karthik/wesanderson) in R.

**Figure 4:**
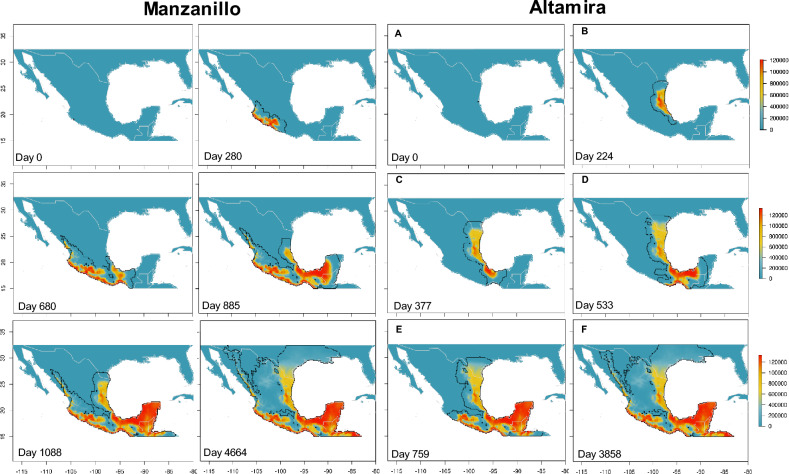
Predicted invasion dynamics and abundance of the ambrosial complex *X. bispinatus*–*H. lauricola* from the port of Manzanillo and Altamira at different time periods. Maps were generated with the raster package ver. 3.6 and wesanderson color palette (https://github.com/karthik/wesanderson) in R.

**Figure 5:**
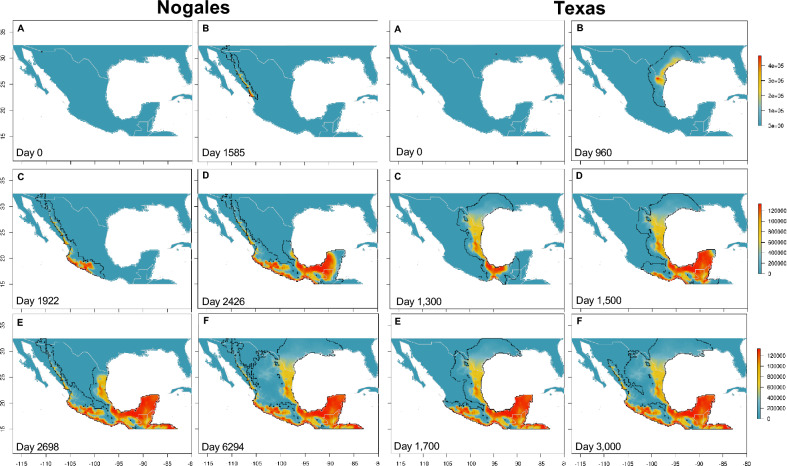
Predicted invasion dynamics and abundance of the ambrosial complex *X. bispinatus*–*H. lauricola* from the port of Nogales and Texas at different time periods. Maps were generated with the raster package ver. 3.6 and wesanderson color palette (https://github.com/karthik/wesanderson) in R.

The states of Veracruz, Chiapas, Oaxaca, and the Isthmus of Tehuantepec (including Tabasco) and the central avocado region of the country were the most threatened areas requiring monitoring for known or secondary vectors and the disease (also see synthesis of the simulations dynamics in Supplementary Table S2). Different spread speeds were observed in the invasion dynamics depending on the starting points of the simulation, the fastest being those that began from the ports of Salina Cruz and Veracruz, which took approximately 650 days to cover these areas (Fig. 3), followed by the ports of Manzanillo and Altamira with 760 and 970 days, respectively (Fig. 4). In Manzanillo, the invasion of the avocado area was the most important and rapid, completed within 350 days. In the case of Nogales customs, the elapsed time to cover these areas was 2670 days, while from Texas (a site where *X. glabratus* and *X. bispinatus* coexist and *H. lauricola* is known to be present) was 1700 days (Fig. 5). Finally, the simulation from the port of Ensenada in Baja California did not prosper due to a lack of suitable sites (see all simulations in Supplementary Videos online).

## Discussion

### Optimal growth temperatures, maximum fitness and growth function

Insects are susceptible to thermal variation^121–129^. In artificially reared Scolytinae beetles, temperature has been documented to be a key factor for their physiology, abundance, and distribution^130–138^ as well as that of their fungal symbionts^67,139,140^. Here, rearing of *X. bispinatus* yielded similar results in terms of demographic parameters relative to its artificially reared relatives at comparable temperatures^132,134,141–145^. Our experiment showed the highest population growth between 26 and 29 °C. This optimal range was also recorded in *X. affinis*, another native secondary vector that was reared following a similar methodology^136^ and had substantial population growth between 20 and 30 °C^146,147^. In *X. glabratus,* Brar et al.^132^ reported that the species completed its life cycle successfully at 24, 28 and 32 °C, with the highest rate of oviposition and development at 28 °C, and suggest through linear models that the lowest threshold for eggs and pupae would be at 13.8 °C and 11.1 °C, respectively. Likewise, these authors^132^ reported that the rate of development observed for *X. glabratus* is similar to that reported in the literature for *X. fornicatus* and other Scolytinae species.

Although best growth for *X. bispinatus* occurred at 26 and 29 °C in the laboratory, the adjustment of the growth curve indicates an optimal temperature ${t}_{opt}$ at 26.26 °C, which might imply optimal breeding temperatures between 26 and 27 °C. Such values, however, were derived from individuals obtained from a population of Los Tuxtlas, Veracruz (Fadda et al., in review), and we should assume conservatism in this abiotic dimension of their fundamental ecological niche for extrapolation^25,115,148–151^; this is reinforced by data on the thermal niche of phylogenetically related species^152–156^. This implies that it would be possible to use demographic data of phylogenetically and functionally close species of *Xyleborus* as surrogates of their growth rates when there are phytosanitary restrictions for their direct use in the field or in laboratories that do not have the minimum necessary security levels^104,157^.

### Mapping of intrinsic growth rate and influence of internal structure of the niche

Our methodological proposal suggests the use of demographic information from which optimal growth rates can be derived as a function of an abiotic factor (temperature in this case) and its expression in the geography (*r*_*i*_ map). The *r*_*i*_ map represents the relationship between the growth rate and the internal structure of the niche projected in geographic space^11,17^, thus indicating the distribution of hierarchies of climatic importance for the species where the process-explicit model will run. The *r*_*i*_ map showed that the greatest demographic growth coincides with sites of greatest diversity of Lauraceae species^40^, including the municipalities of avocado production^94^ (SIAP; http://infosiap.siap.gob.mx/gobmx/datosAbiertos.php). This is a consistent result with what Lira-Noriega et al*.*^63^ found using correlative ecological niche models for Lauraceae and *Xyleborus* species, with the neotropical region being the most vulnerable to the invasion of *X. glabratus* and *Euwallacea* spp., and for the distribution of *X. bispinatus*. Therefore, considering and incorporating the suitability expressed in the *r*_*i*_ map as a function of one (e.g., temperature) or more dimensions of the ecological niche provides useful and pragmatic information that could be considered in decision-making for managing harmful species. Characterization the intrinsic growth rate is fundamental for forecasting future population trends^32^.

There are few antecedents about the use of species’ growth thresholds and physiology-based correlative models in *Xyleborus*^158^. Most of this approach has focused on beetles of the genera *Dendroctonus*, *Hylobius*, *Ips*, and *Hypothenemus*^68^, which utilize phenological models integrating degree days to estimate the number of annual generations and predict population outbreaks of beetles. However, these models do not incorporate the upper and lower growth thresholds in these species, which poses a limitation when making predictions under changing climate scenarios^159^. An alternative approach that predicts this effect and uses correlative models is exemplified by the proposal of DeRose et al*.*^71^, who estimated the impact of increasing current and future temperatures on the distribution of *D. rufipennis* with BIOMOD. Evangelista et al.^61^ used Maxent and Duehl et al*.*^69^ CART (classification and regression tree) in *D. ponderosae* and *D. frontalis*, respectively, to estimate the suitability and importance of factors determining their distribution in North America. Jaramillo et al*.*^70^ incorporated physiological data and degree days using CLIMEX to estimate the consequences of climate change on the number of generations of *H. hampei* (coffee berry borer) worldwide. These studies suggest that implementating process-explicit modeling represents a significant advancement in studying patterns and processes that influence the distribution of organisms with similar characteristics and of phytosanitary relevance.

### Metapopulation process-explicit model

The implemented metapopulation process-explicit model, sometimes also referred to as hybrid models or coupled niche-population models^48^, combines information derived from correlative models with demographic and dispersal parameters to predict abundances and trajectories through a deterministic process. This model enabled us to test potential dispersal routes from multiple starting points across a large geographic extent. Although the predictions of our process-explicit simulations largely coincide with the suitabilities predicted by the correlative model, despite being initiated at different ports and customs across Mexico, they are useful for explaining likely patterns of movement across the territory of the ambrosia beetles (see Supplementary Videos online). The highest risk occurs if the simulation starts from Salina Cruz and Veracruz, probably due to the high environmental suitability and geographic proximity to the Isthmus of Tehuantepec and the avocado area and decreases in regions of northern Mexico with less suitability and connectivity (Fig. 3). Despite simulations initiated at Altamira and Manzanillo showing a slowdown in dispersion, their proximity to important avocado planting areas warrants monitoring (Fig. 4; Supplementary Table S2 online). The simulations from Nogales and from Texas take longer to arrive at highly suitable areas given the low intrinsic growth rates. However, from these sites the ambrosia beetles would be able to reach suitable sites such as Tamaulipas in the Gulf of Mexico or Jalisco in the Pacific (Fig. 5) from where a large expansion could occur in the interior of Mexico. Finally, the simulation from Ensenada did not prosper due to the low suitability, thus limiting the invasion progress to other regions.

The use of actual or hypothetical dispersal rates and of sites chosen for initiating the process-explicit simulations (e.g., ports, customs, or natural or human-facilitated dispersal rates) are helpful in assessing different invasion routes and the speed at which they can occur. This highlights the importance of connectivity corridors (Supplementary Video online). Additionally, we were able to identify source and sink sites/populations, which are often difficult to identify without estimations of population growth rates and environmental suitability throughout their distribution^13,30,160,161^. For our specific case, we assumed a constant dispersal rate of 5 km per day and a carrying capacity of 130,000 individuals per cell as hypothetical density dependency. However, such parameters could be adjusted based on available data from field or laboratory experiments or from rates reported in the literature. For example, Koch et al*.*^162^ used a dispersal rate for *X. glabratus* in the USA of 54.8 km/year (approximately 0.15 km/day), which they estimated as the linear distance between infected counties between two years. Even though the dispersal rate estimated in this paper is hypothetical, determining the true dispersal of these ambrosia beetles in the field is difficult, and there is currently no information available regarding this aspect for *X. bispinatus*^163^. However, process-explicit models should allow for the evaluation of multiple dispersal rates/scenarios, perhaps due to the influence of anthropogenic interference and facilitation^53,63^.

### Limitations and new horizons for the modeling of species niches and distributions

This study represents an effort to develop a tool based on ecological niche theory and process-explicit models that contribute to understanding the mechanisms and patterns responsible for the distribution of organisms. Despite illustrating its use in invasive species, its application can be extended to the study of other kinds of organisms and species assemblages at multiple scales in space and time^53^. The key advantage of process-explicit models lies in the capability of explicitly incorporating ecological mechanisms to better understand the determinants of species distributions^53,164^. However, the development of a process-explicit model depends primarily on the biological assumptions that are being explored and the mathematical form of the model. Some limitations of metapopulation models, such as the one implemented in this work, may include the mathematical model not being the most appropriate, challenges in obtaining demographic parameters, dispersion rates, and density-dependency factors, or inherent errors in the correlative models^54^ used as input for estimating suitability and the map of the intrinsic rate of growth (*r*_*i*_)^48^.

Most of the studies on biological invasions show the use of reaction–diffusion models that assume that space is continuous^165^. In contrast, our model uses discrete space, allowing for consideration of heterogeneous environments and the presence of dispersal barriers. In our approach, this can be addressed by assigning different dispersal rates (e.g., setting the dispersal rate between patch *i* and patch *h* to zero; ${\delta }_{ih}=0$). A relevant factor not included in our study is the Allee effect, which plays a significant role as it emphasizes how regions of low suitability can act as biological barriers to dispersion^19,165,166^, and influence the temporal trajectory of invasion spread or population expansion. Moreover, Osorio-Olvera et al*.*^19^ demonstrated that strong Allee effects can obscure the relationship between population abundance and niche structure because migration fails to start a population below an Allee threshold. However, this effect is less pronounced in our case because the reproductive system of *X. bispinatus* is not affected by causes of inbreeding, and there is a low energy cost for finding reproductive partners^167–169^. Therefore, while the Allee effect may not be as relevant in the specific case of *Xyleborus* species due to their capacity for sib-mating or arrhenotokous inbreeding^67^, it is crucial to recognize its potential impact on other taxonomic groups that rely on it whenever this methodology and simulations are employed. Given the challenges associated with parameterizing demography-dependent process-explicit models, an alternative could be cellular automata models, where system states are presence/absence and a binarized niche model along with a first-neighbor dispersal kernel would suffice to estimate invasion paths^164^. However, these models cannot capture the level of complexity that process-explicit models like ours do, which incorporate demography and other key factors in many ecological and evolutionary processes.

We acknowledge that obtaining demographic data can be complex, especially for cryptic species that are challenging to study due to their complex behavior or lifestyle. Additionally, it may not always be feasible to follow cohorts. One way to overcome such limitation is through laboratory measurements where environmental conditions are controlled, although these come to the expense of not always reflecting the reality of the species’ environment and the ways in which it affects its demography. Another limitation is related to the influence of several environmental dimensions on large-scale interpopulation variations. This might imply that demographic parameters estimated from one population may not be representative of the entire species^33^. Furthermore, the weighting of a few locally adapted units may underestimate the species’ ecological niche^3^, especially if we lack information on whether these are source or sink populations^13,30,51,160^. However, laboratory studies and experiments are currently the most widely used methods in studying *Xyleborus*^108,130,134,136^ and are considered a superior method for the study of ambrosia beetles^132^. This gains more importance considering that laboratory studies may be the only alternative for obtaining demographic information compared to other tools, such as COMPADRE and COMADRE (https://compadre-db.org/Data), which may lack data for certain taxa and areas where they are distributed.

With regard to the use of correlative models of ecological niche, errors or biases can arise from various sources, including the quality of presence records (e.g., spatial, taxonomic, etc.), overall model quality, and the predicted suitability. Similarly, the scale and extrapolations in time and space can lead to over- or under-estimations in these predictions^48,170,171^. We consider that these potential weaknesses could be mitigated because the training and validation of our model was carried out on a species within its native range under current climatic conditions. We also took careful consideration of errors on the input data and thoroughly evaluated the model. Furthermore, there is potential for improvement by incorporating microclimatic variables, which may be extremely important for the physiology of these species and their symbionts^67,172–174^. Despite the potential drawbacks associated with the use of our process-based model, it remains a promising tool in ecology and evolution that could aid in better understanding species distributions.

## Materials and methods

### Collection of individuals in the field, experimental laboratory conditions and estimation of the growth rate at different temperatures

#### Collection of individuals of *X. bispinatus*

Individuals were collected using various methodologies: infested trunks, females in flight, and bottle traps at the Estación de Biología Tropical of the Universidad Nacional Autónoma de México in the state of Veracruz. They were then transported to the Laboratorio de Entomología Molecular of the Instituto de Ecología, A.C., where they were conditioned and maintained in artificial culture media^141^ at 26 °C and 60% relative humidity. The colony was maintained under these conditions until reaching the third filial generation in the laboratory, where live females were meticulously chosen for the experiment.

#### Culture medium

We used a modified culture medium of Biedermann et al*.*^141^: 45 g beech sawdust (*Platanus mexicanus*), 12 g agar, 6 g sucrose, 3 g casein, 3 g starch, 3 g yeast, 0.6 g Wesson's salt mixture, streptomycin 0.21 g, 1.5 mL of wheat germ oil, 3 mL of 96% ethanol, and 400 mL of distilled water. The preparation was carried out in glass bottles of 1 L capacity. The final mixture was sterilized in an autoclave at 121 °C and 15 PSI for 20 min. Using a laminar flow hood, the sterile medium was poured into 50 mL Falcon ™ polypropylene tubes until completing 15 mL, allowing it to dry for 12 h. Finally, the tubes were kept at 26 °C and 60% relative humidity until the day of the experiment.

#### Experimental rearing conditions

In Scolytinae with haplodiploid reproduction, adults can mate with siblings within the natal nest before dispersal. Based on this biological characteristic, we assume that all the females used for the experiment were fertilized^130,175,176^. Prior to inoculation, *X. bispinatus* females were sterilized by immersion for 5 s in 70% alcohol, and later, in water for the same time to remove the alcohol. Surviving females were individually inoculated into 50 mL Falcon™ tubes containing culture medium. To favor gas exchange and prevent individuals from escaping, the tube caps were perforated and covered with a metal mesh. The inoculated tubes were placed in rearing chambers at constant temperatures of 17, 20, 26, 29, and 35 °C, arranged vertically and kept in complete darkness (Supplementary Fig. S1 online). Each temperature treatment included 90 tubes, resulting in a total of 450 inoculated tubes for the entire experiment. Temperature monitoring in each growth chamber was performed using the Elitech URC-4 device.

#### Experimental count of individuals

Every four days (throughout 36 days that the experiment lasted), 10 tubes from each rearing chamber temperature were checked. The artificial medium was carefully dissected (destructive monitoring) and the number of eggs, larvae, pupae and adults (males and females; Supplementary Fig. S2 online) was counted. In addition, for this last stage of development, living and dead individuals were recorded. Observations were made with a Leica EZ4 stereomicroscope. The experiment took place over 36 days, totaling nine colony monitoring openings. Individual counts at each colony opening date were averaged and accumulated. With this final accumulated average of the experiment for each stage of development and temperature, the biological parameters of the species were estimated.

#### Egg hatching percentage

Accounting for hatched eggs is extremely important when estimating the growth rate of the species. However, due to the cryptic habit of *X. bispinatus*, which requires destructive monitoring of the culture medium, it was not possible to make such an estimate. To correct this, we used information from *Dendroctonus ponderosae,* a species related to the genus *Xyleborus*^79,102,177^.We consider that this procedure represents a more viable alternative than assuming a 100% hatching rate. The use of proxies or assumptions to obtain limited demographic information is a valid method, especially when it is necessary to make management decisions before they can be collected^178,179^. Furthermore, it is important to highlight that *Xyleborus bispinatus* present similar patterns regarding optimal growth and the upper and lower population thresholds (Fadda et al*.*, in review) as *D. ponderosae* when raised at similar laboratory temperatures^180,181^, thus enhancing the robustness of our methodological approach.

The information regarding the percentages of hatched eggs was obtained from mountain pine beetles (*Dendroctonus ponderosae*) reared axenically on a standardized diet at constant temperatures of 10, 18, 20, 24, 27, 32, and 35 °C^180^. Using this information, we fitted a second-order polynomial curve using the nls.lm function of the minpack.lm R package^182,183^. From this curve, we obtained an estimation of the egg hatching percentage equivalent to the temperatures tested in our work. Subsequently, these values were used in conjunction with the number of egg laying made by *X. bispinatus* to estimate the net reproductive rate necessary to calculate the growth rate of the species at its different temperatures (Eq. 1; Supplementary Fig. S3 online).

1 $$p\left(T\right)=a{T}^{2}+bT+c$$

where $p$ = Proportion of hatched eggs, *T* = Temperature, *a, b* and *c* = parameters of the regression.

#### Survival of developmental stages

Survival (${S}_{k}$; Eq. 2) was calculated for each of the stages of the life cycle of *X. bispinatus* (see section "Experimental count of individuals"). This is computed by the quotient of the individuals belonging to the immediate following stage of development to the one of interest (*k* + 1) and the value obtained for the stage that we wish to estimate (*k*). The equation returns values between zero and one, and is estimated as follows:

2 $${S}_{k}=\frac{{N}_{k+1}}{{N}_{k}}$$

where ${S}_{k}$= Survival at development stage *k*, ${N}_{k+1}$= Number of individuals at stage $k+1$, ${N}_{k}$= Number of individuals at development stage of interest.

In the case of eggs, the hatching percentage at each temperature was first estimated (Eq. 1) and then the survival rate. In adults, survival was only estimated for females since they are mainly responsible for the population growth of the species^67^. The number of individuals was estimated from the quotient between the total number of females (live and dead) and the values of live females recorded in the experiment.

#### Net reproductive rate

The net reproductive rate (${R}_{0}$; Eq. 3) was estimated according to what was stipulated by Southwood^184^ as the product of survival in each stage of development and the proportion of hatched eggs estimated in section "Egg hatching percentage" at each temperature:

3 $${R}_{0}=pH\prod_{k=1}^{n}{S}_{k}$$

where ${R}_{0}$= Net reproductive rate, *p* = Proportion of hatched eggs, $H$= Number of eggs counted, *S*_k_ = Survival rate at stage *k.*

#### Population intrinsic growth rate

The intrinsic growth rate of the population ($\normalsize {r}$; Eq. 4) was estimated as the quotient between the natural logarithm of the net reproductive rate and the generation time ($G$) observed at the temperature of interest. $G$ is defined as the time between the first observation of the egg stage and the first count of an adult hatchling of the progeny in question at a given temperature:

4 $$r=\frac{ln\left({R}_{0}\right)}{G}$$

where $\left(r\right)$ = Population intrinsic growth rate, ${R}_{0}$= Net reproductive rate at a given temperature, $G$ = Generation time, days from the first observation of eggs to the first record of females from the progeny.

#### Function of intrinsic growth rate

We fitted a non-linear model to the values of the intrinsic growth rate obtained for each temperature (section "Population intrinsic growth rate"), using the nls.lm function of the minpack.lm R package. This was based on a convex mathematical function (Eq. 5) whose parameters are biologically meaningful: an optimum suitability for the species (centroid), expressed in terms of optimum temperature $\left({t}_{opt}\right)$ where the maximum intrinsic growth rate of *X. bispinatus* occurs $\left({r}_{max}\right)$, and the kurtosis of the fitted curve (${v}_{o}$; a positive real number that determines the niche breadth):

5 $$r\left(T\right)=\frac{{r}_{max}}{exp\left({v}_{0}{\left(T-{T}_{opt}\right)}^{2}-{v}_{0}\left(T-{T}_{opt}\right)\right)}$$

$r$ = Intrinsic growth rate, ${r}_{max}$= Maximum intrinsic growth rate, $T$= Temperature, ${T}_{opt}$= Optimum growth temperature, ${V}_{0}$= Kurtosis of fitted curve.

### Ecological niche modeling

To mitigate the fact that we modeled the intrinsic growth rate as a function of one variable (temperature) and this parameter might depend on other factors such as humidity^32^, we applied a two-step modeling approach before evaluating the intrinsic growth rate in the geographic space. First, we employed correlative niche models fitted using bioclimatic variables to consider the effect of seasonal variations of precipitation and temperature on the potential distribution of this species; the above allowed us to delimit the sites where the evaluation of *r* made biological sense^185^. Then, we evaluated the function that relates the mean temperature with the intrinsic growth rate (Eq. 5) on those sites with suitable conditions of precipitation and temperature.

#### Occurrences

We compiled georeferenced and dated records of *X. bispinatus* in America from: BarkBeetles (https://www.barkbeetles.info/), the Global Biodiversity Information Facility (GBIF; https://www.gbif.org/)^186^, bibliographic review of scientific journals, and collected specimens at Los Tuxtlas, Veracruz. With Google Earth Pro 7.3, we verified these records coincided with field collections and discarded records from cities, museums, and other scientific collections. Additionally, we eliminated temporal (same year) and spatial (at 30 arc seconds) duplicate records. This produced a total of 106 presence points for the species of which 70% were used for calibrating the model and 30% for its validation from a random partition.

#### Environmental variables and time specific niche modeling

Monthly climatic data on minimum temperature, maximum temperature and cumulative precipitation were obtained from the CHELSAcruts database^187,188^ at a resolution of 30 arc seconds for the period comprising 1986–2016. With this information and following the methodology in O'Donnell and Ignizio^189^, bioclimatic layers were constructed for each year during the period in question.

To estimate the ecological niche of *X. bispinatus*, we used time-specific niche models (also known as time calibrated species distribution models^190^). This approach allows us to calibrate niche models with spatial information of high temporal resolution, reducing niche and distribution estimation biases^191^. The modeling algorithm we employed was minimum volume ellipsoids, as they are a simple and biologically informative way to represent an *n*-dimensional hypervolume according to the original proposal of Hutchinson^11^. This proposal considers a niche to have a convex shape where the maximum fitness is at the centroid of the ellipsoid and decreases towards the periphery^16^. Moreover, it has been documented that the distance to the center of an ellipsoid is related to fitness attributes, such as population abundance and genetic diversity^192,193^.

The modeling process was carried out using the tenm (temporal ecological niche models)^194^ package in R^183^, which allows a time-specific model selection process to be executed. In this process, the data were first thinned using a distance of 0.0083 degrees to reduce problems associated with spatial autocorrelation. Likewise, the environmental information corresponding to the year of the presence records was extracted and the Pearson correlation coefficient was estimated; those whose correlation was less than 0.8 were chosen as modeling variables. The selection of better models was done considering a significance of 95% for the partial ROC statistical test^195^(1000 iterations and 50% of the data was used for the bootstrap), in addition, the partial AUC ratio and AUC value were calculated from 50,000 randomly selected environment points, a performance criterion of 10% omission and with the lowest environment prevalence percentage to decrease the potential error of commission and thus avoid overestimation; this selection process has been used in other modeling works^192,196,197^. Finally, these were converted to a binary prediction (presence-absence) based on a 10th percentile threshold.

#### Adjusted growth map from the intrinsic growth rate function

Prior to running the models, we designed as the calibration area^185^ the geographical region encompassing the Mexican territory together with the sympatry zone shared by the species *X. glabratus* and *X. bispinatus* in the United States, given that the first might be infected by the last. Then, in order to perform the metapopulation process-explicit dynamic models, we estimated the growth rate in the study area by evaluating Eq. 5 on each pixel with the annual mean temperature layer (2.5' resolution; ~ 5 km per pixel) from WorldClim^120^; this produced a *r*_*i*_ map. To consider the effect of other environmental variables such as precipitation on growth rate and to reduce the overprediction related to some sites having temperature values outside the fundamental niche of *X. bispinatus* at certain times of the year, the *r*_*i*_ map was multiplied by the binary suitability map of the final model. Finally, with the crop and mask functions of the raster R package^198^, we proceeded to delimit the polygon where the simulation was carried out.

#### Modeling the invasion dynamics of *Xyleborus bispinatus*

The spatiotemporal dynamics of *X. bispinatus* was estimated as a function of climatic conditions, growth rate and density dependence factors. For this, the metapopulation model of Eq. 6 was used. In this model it was assumed that the species grows in a region divided by a regular grid with con $i=1, 2\dots n$ number of cells. In the absence of migratory processes, the growth of a population ${x}_{i}$ is determined by its biology and the environmental characteristics of cell $i$. During the invasion process, the populations ${x}_{i}$ are connected by dispersal, therefore, the population abundance in each cell $i$ also depends on the rates of entry and exit of individuals in cells $i$. The metapopulation model is shown below^16^

6 $${\dot{x}}_{i}={r}_{i}{x}_{i}-{a}_{i}{x}_{i}^{2}+{\sum }_{h}{\delta }_{hi}{x}_{h}-{\sum }_{i}{\delta }_{ih}{x}_{i}$$

where ${\dot{x}}_{i}$ is the rate of population change in cell $i$ at time $t$. ${r}_{i}$ is the intrinsic growth rate of cell $i$ which was obtained from the growth rate map for the zone of interest. $a$ is a dense-dependence factor which measures the intensity of intraspecific competition. ${\delta }_{hi}$ is the immigration rate from cells $h$ to cell $i$; ${\delta }_{ih}$ is the migration rate from cell $i$ to the other cells. The immigration and migration rates are determined by a distance-dependent first-neighbor dispersal kernel (Eqs. 7A and 7B). This procedure composes a quadratic system of coupled nonlinear differential equations that requires solving as many equations as the number of cells, which implies a relevant processing time as well as a large computational capacity (deciphering approximately 63,000 equations at the resolution and spatial extent worked). A resolution of 2.5 min per cell was considered for the simulation in the calibration area, since at finer resolutions the numerical solution of the model was not possible.

The value of the density dependence parameter *a* was set at $a$ = 0.0000001, to produce population densities (in units of individuals per km^2^) of approximately 130,000 individuals in the most suitable cells (considering that the carrying capacity in the model without dispersion is $\frac{{r}_{max}}{a}$).

$${\delta }_{ih}\left({w}_{ih}\right)={\delta }_{hi}\left({w}_{hi}\right)=\left\{\begin{array}{c}\begin{array}{c} \\ k exp\left[{-w}_{hi}^{b}\right]\end{array}\\ 0\end{array}\right. \begin{array}{c} {w}_{hi} \le {D}_{max} \quad(7{\text{A}})\\ { w}_{hi} >{D}_{max} \quad(7{\text{B}}) \end{array}$$

#### Dispersion kernel

To model dispersal between cells, we used an exponential kernel^50,199^ where ${D}_{max}$ is the maximum distance an individual can travel per day between patches, and was established at ~ 5 km^67,162,163,200,201^; ${\delta }_{ih}$ and ${\delta }_{hi}$ are migration rates of individuals between cells; $k$ is the maximum migration capacity, which was set as one tenth of the intrinsic growth rate *r*_*i*_ and $b$ is a positive constant that modulates travel capacity. The distance between cells was measured with Euclidean distance using the cell coordinates $h$ and $i$. The barriers that prevent migration from patch $h$ to patch $i$ are simulated by taking ${\delta }_{hi}= {\delta }_{ih}=0$. In our case we relied on an irreducible dispersal matrix; that is, there is the possibility of reaching all grid elements, from any other cell including those of low suitability and without taking into consideration biotic barriers such as the Allee effect^19^.

#### Starting points for modeling invasion routes

**Figure 6:**
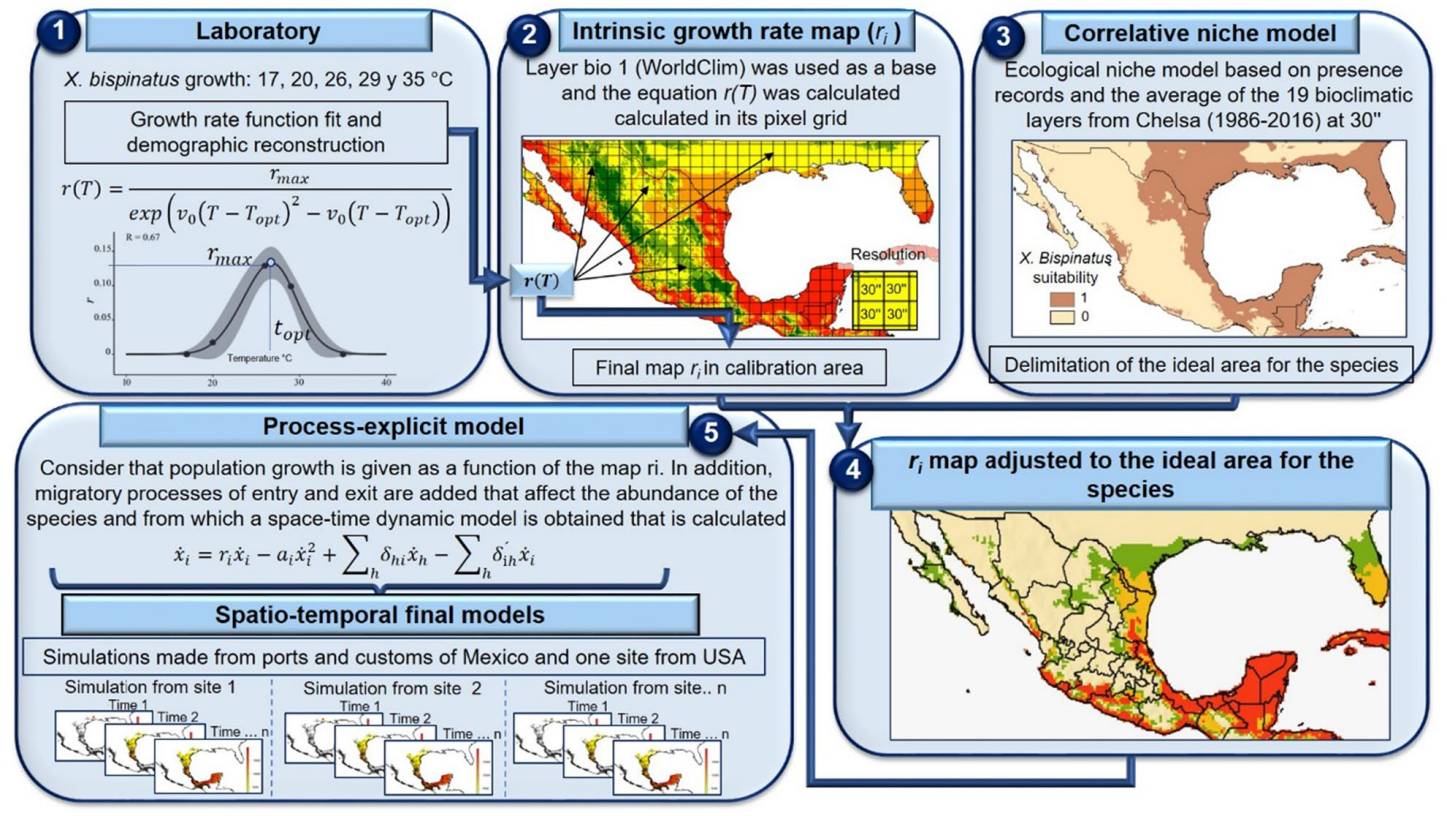
Workflow diagram used for the construction of the process-explicit model simulations. The figure was assembled with the growth curve depicted in Fig. 1, along with maps created using the raster package version 3.6 in R and ArcGIS version 10.4. (https://www.arcgis.com/features/index.html).

We defined various starting points in Mexico to conduct the simulations, including ports and customs. This decision was primarily influence by the fact that the great majority of ambrosia beetles are favored by anthropogenic dispersal, which facilitates their escape from natural barriers that might otherwise confine them to their native ranges. Thus, we considered the geographic coordinates for the ports of Salina Cruz, Veracruz, Manzanillo, Altamira and Ensenada, and the land customs of Nuevo Laredo and the Nogales. In addition, we also considered a site in Texas where *X. bispinatus* and *X. glabratus* are geographically close and the disease is known to be present, representing a higher risk for Tamaulipas as a potential gateway through which the vector and the pathogen could enter Mexico by land (Supplementary Table S1 online). The summary diagram of the workflow carried out in this study is shown in Fig. 6.

### Ethics approval and consent to participate

This article does not describe any studies involving human participants performed by the authors. All applicable international, national and/or institutional guidelines for the care and use of animals were followed.

### Supplementary Information

Supplementary Video 1.

Supplementary Information 1.

Supplementary Video 2.

Supplementary Video 3.

Supplementary Video 4.

Supplementary Video 5.

Supplementary Video 6.

Supplementary Video 7.

Supplementary Information 2.

Supplementary Information 3.

Supplementary Information 4.

## Data Availability

All supplementary material is fully available in the figshare repository [10.6084/m9.figshare.24915975.v4].

## References

[1] Sagarin RD, Gaines SD, Gaylord B (2006). Moving beyond assumptions to understand abundance distributions across the ranges of species. Trends Ecol. Evol..

[2] Van Der Have, T. M. A proximate model for thermal tolerance in ectotherms. *Oikos*, **98**, 141–155 (2002).

[3] Peterson AT, Papeş M, Soberón J (2015). Mechanistic and correlative models of ecological niches. Eur. J. Ecol..

[4] Elton, C. S. *The Ecology of Invasions by Animals and Plants.* (1958). T. Methuen and Co., London.

[5] Skellam, J. G. Random dispersal in theoretical populations. *Biometrika* **38**, 196–218 (1951). 14848123

[6] Fisher, R. A. The wave of advance of advantageous genes. *Ann. Eugen.* **7**, 355–369 (1937).

[7] Hanski I (1998). Metapopulation dynamics. Nature.

[8] Hanski I (1999). Metapopulation Ecology.

[9] Okubo A, Levin SA (2001). Diffusion and Ecological Problems: Modern Perspectives.

[10] Pielou EC (1969). An Introduction to Mathematical Ecology.

[11] Hutchinson GE (1957). Concludig remarks. Cold Spring Harb. Symp. Quant. Biol..

[12] Haby NA (2013). Scale dependency of metapopulation models used to predict climate change impacts on small mammals. Ecography (Cop.).

[13] Pulliam HR (2000). On the relationship between niche and distribution. Ecol. Lett..

[14] Soberón J (2010). Niche and area of distribution modeling: A population ecology perspective. Ecography (Cop.).

[15] Soberón J (2007). Grinnellian and Eltonian niches and geographic distributions of species. Ecol. Lett..

[16] Osorio-Olvera LA, Falconi M, Soberón J (2016). Sobre la relación entre idoneidad del hábitat y la abundancia poblacional bajo diferentes escenarios de dispersión. Rev. Mex. Biodivers..

[17] Maguire B (1973). Niche response structure and the analytical potentials of its relationship to the habitat. Am. Nat..

[18] Martínez-Meyer E, Díaz-Porras D, Peterson AT, Yáñez-Arenas C (2013). Ecological niche structure and rangewide abundance patterns of species. Biol. Lett..

[19] Osorio-Olvera LA, Soberón J, Falconi M (2019). On population abundance and niche structure. Ecography.

[20] Elith J (2006). Novel methods improve prediction of species’ distributions from occurrence data. Ecography (Cop.).

[21] Soberón J, Nakamura M (2009). Niches and distributional areas: Concepts, methods, and assumptions. Proc. Natl. Acad. Sci. USA.

[22] Anderson RP, Lew D, Peterson AT (2003). Evaluating predictive models of species’ distributions: Criteria for selecting optimal models. Ecol. Modell..

[23] Peterson AT (2011). Ecological Niches and Geographic Distributions.

[24] Jiménez L, Soberón J, Christen JA, Soto D (2019). On the problem of modeling a fundamental niche from occurrence data. Ecol. Modell..

[25] Peterson AT (2011). Ecological niche conservatism: A time-structured review of evidence. J. Biogeogr..

[26] Newman JC, Riddell EA, Williams LA, Sears MW, Barrett K (2022). Integrating physiology into correlative models can alter projections of habitat suitability under climate change for a threatened amphibian. Ecography.

[27] Cuervo-Robayo AP (2017). Introducción a los análisis espaciales con énfasis en los modelos de nicho ecológico. Biodivers. Inform..

[28] Cavanaugh KC (2015). Integrating physiological threshold experiments with climate modeling to project mangrove species’ range expansion. Glob. Change Biol..

[29] Dormann CF (2009). Response to comment on ‘methods to account for spatial autocorrelation in the analysis of species distributional data: A review. Ecography..

[30] Schurr FM (2012). How to understand species’ niches and range dynamics: A demographic research agenda for biogeography. J. Biogeogr..

[31] Barve N, Martin C, Brunsell NA, Peterson AT (2014). The role of physiological optima in shaping the geographic distribution of Spanish moss. Glob. Ecol. Biogeogr..

[32] Sibly RM, Hone J (2002). Population growth rate and its determinants: An overview. Philos. Trans. R. Soc. B Biol. Sci..

[33] Chapman DS, Scalone R, Štefanić E, Bullock JM (2017). Mechanistic species distribution modeling reveals a niche shift during invasion. Ecology.

[34] Enriquez-Urzelai E, Kearney MR, Nicieza AG, Tingley R (2019). Integrating mechanistic and correlative niche models to unravel range-limiting processes in a temperate amphibian. Glob. Change Biol..

[35] Kearney M, Porter W (2009). Mechanistic niche modelling: Combining physiological and spatial data to predict species’ ranges. Ecol. Lett..

[36] Valladares F (2014). The effects of phenotypic plasticity and local adaptation on forecasts of species range shifts under climate change. Ecol. Lett..

[37] Birch LC (1953). Experimental background to the study of the distribution and abundance of insects: I. The influence of temperature, moisture and food on the innate capacity for increase of three grain beetles. Ecology.

[38] Hooper HL (2008). The ecological niche of *Daphnia magna* characterized using population growth rate. Ecology.

[39] Hone J (1999). On rate of increase (r): Patterns of variation in Australian mammals and the implications for wildlife management. J. Appl. Ecol..

[40] Etherington TR (2021). Mahalanobis distances for ecological niche modelling and outlier detection: Implications of sample size, error, and bias for selecting and parameterizing a multivariate location and scatter method. PeerJ.

[41] Escobar, L. E. Ecological niche modeling: An introduction for veterinarians and epidemiologists. *Front. Vet. Sci. ***7**, 519050 (2020). 10.3389/fvets.2020.519059 PMC7641643 33195507

[42] Guisan A, Zimmermann NE (2000). Predictive habitat distribution models in ecology. Ecol. Modell..

[43] Veran S (2016). Modeling spatial expansion of invasive alien species: Relative contributions of environmental and anthropogenic factors to the spreading of the harlequin ladybird in France. Ecography (Cop.).

[44] Evans MR (2013). Do simple models lead to generality in ecology?. Trends Ecol. Evol..

[45] De Marco P, Diniz-Filho JAF, Bini LM (2008). Spatial analysis improves species distribution modelling during range expansion. Biol. Lett..

[46] Elith J, Kearney M, Phillips S (2010). The art of modelling range-shifting species. Methods Ecol. Evol..

[47] MacKenzie DI (2017). Occupancy Estimation and Modeling: Inferring Patterns and Dynamics of Species Occurrence.

[48] Briscoe NJ (2019). Forecasting species range dynamics with process-explicit models: Matching methods to applications. Ecol. Lett..

[49] Catterall S, Cook AR, Marion G, Butler A, Hulme PE (2012). Accounting for uncertainty in colonisation times: A novel approach to modelling the spatio-temporal dynamics of alien invasions using distribution data. Ecography (Cop.).

[50] Nenzén HK, Swab RM, Keith DA, Araújo MB (2012). Demoniche: An R-package for simulating spatially-explicit population dynamics. Ecography (Cop.).

[51] Pagel J, Schurr FM (2012). Forecasting species ranges by statistical estimation of ecological niches and spatial population dynamics. Glob. Ecol. Biogeogr..

[52] Cabral JS, Schurr FM (2010). Estimating demographic models for the range dynamics of plant species. Glob. Ecol. Biogeogr..

[53] Pilowsky, J. A., Colwell, R. K., Rahbek, C. & Fordham, D. A. Process-explicit models reveal the structure and dynamics of biodiversity patterns. *Sci. Adv. ***8**, eabj2271 (2022). 10.1126/sciadv.abj2271 PMC9355350 35930641

[54] Hagen O (2022). Coupling eco-evolutionary mechanisms with deep-time environmental dynamics to understand biodiversity patterns. Ecography.

[55] Ingenloff K (2017). Predictable invasion dynamics in North American populations of the Eurasian collared dove *Streptopelia decaocto*. Proc. R. Soc. B Biol. Sci..

[56] Nuñez-Penichet C (2021). Geographic potential of the world’s largest hornet, *Vespa mandarinia* Smith (Hymenoptera: Vespidae), worldwide and particularly in North America. PeerJ.

[57] Palma-Ordaz S, Delgadillo-Rodríguez J (2014). Potential distribution of eight invasive exotic species in the state of Baja California, Mexico. Bot. Sci..

[58] Peterson AT, Robins CR (2003). Using ecological-niche modeling to predict barred owl invasions with implications for spotted owl conservation. Conserv. Biol..

[59] Osorio-Olvera LA (2018). Sobre los mecanismos que determinan la distribución geográfica de una especie: idoneidad del hábitat, dispersión y barreras bióticas.

[60] Ballesteros-Mejia L, Kitching IJ, Beck J (2011). Projecting the potential invasion of the pink spotted hawkmoth (*Agrius cingulata*) across Africa. Int. J. Pest Manag..

[61] Evangelista PH, Kumar S, Stohlgren TJ, Young NE (2011). Assessing forest vulnerability and the potential distribution of pine beetles under current and future climate scenarios in the Interior West of the US. For. Ecol. Manag..

[62] Lestina J (2016). MODIS imagery improves pest risk assessment: A case study of wheat stem sawfly (*Cephus cinctus*, Hymenoptera: Cephidae) in Colorado, USA. Environ. Entomol..

[63] Lira-Noriega A, Soberón J, Equihua J (2018). Potential invasion of exotic ambrosia beetles *Xyleborus glabratus* and *Euwallacea* sp. in Mexico: A major threat for native and cultivated forest ecosystems. Sci. Rep..

[64] Rank A (2020). Risk of the introduction of *Lobesia botrana* in suitable areas for *Vitis vinifera*. J. Pest Sci..

[65] Reyes JA, Lira-Noriega A (2020). Current and future global potential distribution of the fruit fly *Drosophila suzukii* (Diptera: Drosophilidae). Can. Entomol..

[66] Goldberg N, Heine J (2009). A comparison of arborescent vegetation pre- (1983) and post- (2008) outbreak of the invasive species the Asian ambrosia beetle *Xyleborus glabratus* in a Florida maritime hammock. Plant Ecol. Divers..

[67] Vega FE, Hofstetter RW (2015). Bark Beetles: Biology and Ecology of Native and Invasive Species.

[68] Bentz, B. J. & Jönsson, A. M. Modeling bark beetle responses to climate change. In *Bark Beetles: Biology and Ecology of Native and Invasive Species* (Vega F. E. & Hofstetter, R. W.) 533–553. 10.1016/B978-0-12-417156-5.00013-7 (2015)

[69] Duehl A, Bishir J, Hain FP (2011). Predicting county-level southern pine beetle outbreaks from neighborhood patterns. Environ. Entomol..

[70] Jaramillo J (2011). Some like it hot: The influence and implications of climate change on coffee berry borer (*Hypothenemus hampei*) and coffee production in East Africa. PLoS One.

[71] DeRose RJ, Bentz BJ, Long JN, Shaw JD (2013). Effect of increasing temperatures on the distribution of spruce beetle in Engelmann spruce forests of the Interior West, USA. For. Ecol. Manag..

[72] Peterson AT (2003). Predicting the geography of species’ invasions via ecological niche modeling. Q. Rev. Biol..

[73] Hobbs RJ, Humphries SE (1995). An integrated approach to the ecology and management of plant invasions. Conserv. Biol..

[74] Jiménez-Valverde A (2011). Use of niche models in invasive species risk assessments. Biol. Invasions.

[75] Eskalen A (2013). Host range of *Fusarium dieback* and its ambrosia beetle (Coleoptera: Scolytinae) vector in southern California. Plant Dis..

[76] Mendel Z (2012). An Asian ambrosia beetle *Euwallacea fornicatus* and its novel symbiotic fungus *Fusarium* sp. pose a serious threat to the Israeli avocado industry. Phytoparasitica.

[77] Harrington TC, Fraedrich SW, Aghayeva DN (2008). *Raffaelea lauricola*, a new ambrosia beetle symbiont and pathogen on the Lauraceae. Mycotaxon.

[78] Fraedrich SW (2008). A fungal symbiont of the redbay ambrosia beetle causes a lethal wilt in redbay and other Lauraceae in the Southeastern United States. Plant Dis..

[79] Gohli J, Selvarajah T, Kirkendall LR, Jordal BH (2016). Globally distributed *Xyleborus* species reveal recurrent intercontinental dispersal in a landscape of ancient worldwide distributions. BMC Evol. Biol..

[80] Haack RA (2003). Intercepted Scolytidae (Coleoptera) at U.S. ports of entry: 1985–2000. Integr. Pest Manag. Rev..

[81] Haack RA (2006). Exotic bark- and wood-boring Coleoptera in the United States: Recent establishments and interceptions. Can. J. For. Res..

[82] Jordal BH, Beaver RA, Kirkendall LR (2001). Breaking taboos in the tropics: Incest promotes colonization by wood-boring beetles. Glob. Ecol. Biogeogr..

[83] Araújo JPM (2022). Four new species of *Harringtonia*: Unravelling the laurel wilt fungal genus. Fungi.

[84] Evans EA, Crane J, Hodges A, Osborne JL (2010). Potential economic impact of laurel wilt disease on the Florida avocado industry. HortTechnology.

[85] Smith SM, Gomez DF, Beaver RA, Hulcr J, Cognato AI (2019). Reassessment of the species in the *Euwallacea fornicatus* (Coleoptera: Curculionidae: Scolytinae) complex after the rediscovery of the “lost” type specimen. Insects.

[86] Boland JM (2016). The impact of an invasive ambrosia beetle on the riparian habitats of the Tijuana River Valley, California. PeerJ.

[87] Boland, J. M. The Kuroshio Shot Hole Borer in the Tijuana River Valley in 2017–18 (year three): Infestation rates, forest recovery, and a new model. *Final Rep. US Navy, US Fish Wildl. Serv. Southwest Wetl. Interpret. Assoc.* **74**, (2018).

[88] Freeman S (2013). *Fusarium euwallaceae* sp. nov.: A symbiotic fungus of *Euwallacea* sp., an invasive ambrosia beetle in Israel and California. Mycologia.

[89] Crane JH, Peña J, Osborne JL (2008). Redbay ambrosia beetle-laurel wilt pathogen: A potential major problem for the Florida avocado industry. Edis.

[90] García-Avila CDJ (2016). First report of *Euwallacea* nr. *Fornicatus* (Coleoptera: Curculionidae) in Mexico. Florida Entomol..

[91] Morgan AR, Graham K, Green C, Smith-Herron AJ (2017). Distribution of the invasive Redbay Ambrosia beetle *Xyleborus glabratus* in Southeastern Texas. Southwest. Nat..

[92] United States Department of Agriculture. Distribution of counties with laurel wilt disease by year of initial detection. https://ccmedia.fdacs.gov/content/download/95555/file/laurel-wilt-distribution-map.pdf (2022).

[93] Servicio Nacional de Sanidad Inocuidad y Calidad Alimentaria (SENASICA - SADER). Complejo escarabajo ambrosia del laurel rojo *Xyleborus glabratus*-*Raffaelea lauricola*. *Ficha Técnica No. 48* (2019).

[94] La Lorea-Hernández FG (2002). familia Lauraceae en el sur de México: diversidad, distribución y estado de conservación. Soc. Botánica México.

[95] Pérez Silva M (2015). Sinopsis de especies mexicanas del género *Xyleborus* Eichhoff, 1864 (Coleoptera: Curculionidae: Scolytinae). Acta Zoológica Mex..

[96] Pérez-Silva M, Equihua-Martínez A, Atkinson TH (2015). Identificación de las especies mexicanas del género *Xyleborus* Eichhoff, 1864 (Coleoptera: Curculionidae: Scolytinae). Insecta mundi.

[97] Ángel Restrepo, M. Escarabajos ambrosiales y sus hongos simbiontes asociados al cultivo de aguacate “Hass” en Michoacán, México (Universidad Michoacana San Nicolás de Hidalgo, 2019).

[98] Carrillo D (2014). Lateral transfer of a phytopathogenic symbiont among native and exotic ambrosia beetles. Plant Pathol..

[99] Servicio Nacional de Sanidad, Inocuidad y Calidad Agroalimentaria (SENASICA.). Plagas bajo vigilancia activa. https://www.gob.mx/senasica/documentos/plagas-bajo-vigilancia-activa-111260 (2020).

[100] Burns JH, Strauss SY (2011). More closely related species are more ecologically similar in an experimental test. Proc. Natl. Acad. Sci. USA.

[101] Wiens JJ (2010). Niche conservatism as an emerging principle in ecology and conservation biology. Ecol. Lett..

[102] Cognato AI, Hulcr J, Dole SA, Jordal BH (2011). Phylogeny of haplo-diploid, fungus-growing ambrosia beetles (Curculionidae: Scolytinae: Xyleborini) inferred from molecular and morphological data. Zool. Scr..

[103] Robles-Fernández ÁL, Lira-Noriega A (2017). Combining phylogenetic and occurrence information for risk assessment of pest and pathogen interactions with host plants. Front. Appl. Math. Stat..

[104] Robles-Fernández ÁL, Santiago-Alarcon D, Lira-Noriega A (2021). American mammal’s susceptibility to dengue according to geographical, environmental, and phylogenetic distances. Front. Vet. Sci..

[105] Lurgi M, Brook BW, Saltré F, Fordham DA (2015). Modelling range dynamics under global change: Which framework and why?. Methods Ecol. Evol..

[106] Kirkendall LR, Jordal BH (2006). The bark and ambrosia beetles (Curculionidae, Scolytinae) of Cocos Island, Costa Rica and the role of mating systems in island zoogeography. Biol. J. Linn. Soc..

[107] Cruz LF (2018). Developmental biology of *Xyleborus bispinatus* (Coleoptera: Curculionidae) reared on an artificial medium and fungal cultivation of symbiotic fungi in the beetle’s galleries. Fungal Ecol..

[108] Menocal O (2018). *Xyleborus bispinatus* reared on artificial media in the presence or absence of the laurel wilt pathogen (*Raffaelea lauricola*). Insects.

[109] Ploetz RC (2017). Recovery plan for laurel wilt of avocado, caused by *Raffaelea lauricola*. Plant Heal. Prog..

[110] Rabaglia RJ, Dole SA, Cognato AI (2006). Review of American Xyleborina (Coleoptera: Curculionidae: Scolytinae) occurring north of Mexico, with an illustrated key. Ann. Entomol. Soc. Am..

[111] Schedl KE (1962). Scolytidae and Platypodidae Afrikas. Rev. Entomol. Moçambique.

[112] Wood SL (1982). The Bark and Ambrosia Beetles of North and Central America (Coleoptera: Scolytidae), a taxonomic monograph. Gt. Basin Nat. Mem..

[113] Faccoli M, Campo G, Perrotta G, Rassati D (2016). Two newly introduced tropical bark and ambrosia beetles (Coleoptera: Curculionidae, Scolytinae) damaging figs (*Ficus carica*) in southern Italy. Zootaxa.

[114] Ploetz RC (2017). Presence and prevalence of *Raffaelea lauricola*, cause of laurel wilt, in different species of ambrosia beetle in Florida, USA. J. Econ. Entomol..

[115] Wiens JJ, Graham CH (2005). Niche conservatism: Integrating evolution, ecology, and conservation biology. Annu. Rev. Ecol. Evol. Syst..

[116] Simberloff D (2013). Impacts of biological invasions: What’s what and the way forward. Trends Ecol. Evol..

[117] Vilà M (2011). Ecological impacts of invasive alien plants: A meta-analysis of their effects on species, communities and ecosystems. Ecol. Lett..

[118] Hulme PE (2009). Trade, transport and trouble: Managing invasive species pathways in an era of globalization. J. Appl. Ecol..

[119] Hulme PE (2010). Biosecurity: The changing face of invasion biology. Fifty years’ invasion Ecol. Leg. Charles Elt..

[120] Hijmans RJ, Cameron SE, Parra JL, Jones PG, Jarvis A (2005). Very high resolution interpolated climate surfaces for global land areas. Int. J. Climatol..

[121] Colinet H, Sinclair BJ, Vernon P, Renault D (2015). Insects in fluctuating thermal environments. Annu. Rev. Entomol..

[122] Foden WB (2019). Climate change vulnerability assessment of species. Wiley Interdiscip. Rev. Clim. Change.

[123] Gunderson AR, Stillman JH (2015). Plasticity in thermal tolerance has limited potential to buffer ectotherms from global warming. Proc. R. Soc. B Biol. Sci..

[124] Kellermann V, Sgrò CM (2018). Evidence for lower plasticity in CTMAX at warmer developmental temperatures. J. Evol. Biol..

[125] Rodrigues YK, Beldade P (2020). Thermal plasticity in insects’ response to climate change and to multifactorial environments. Front. Ecol. Evol..

[126] Angilletta, M. J. Thermal adaptation: A theoretical and empirical synthesis. 289 Oxford University Press. 10.1093/acprof:oso/9780198570875.001.1 (2009).

[127] Knies JL, Kingsolver JG (2010). Notes and comments erroneous Arrhenius: Modified Arrhenius model best explains the temperature dependence of ectotherm fitness. Am. Nat..

[128] Deutsch CA (2008). Impacts of climate warming on terrestrial ectotherms across latitude. Proc. Natl. Acad. Sci. USA.

[129] González-Tokman D (2020). Insect responses to heat: Physiological mechanisms, evolution and ecological implications in a warming world. Biol. Rev..

[130] Biedermann PHW (2010). Observations on sex ratio and behavior of males in *Xyleborinus saxesenii* Ratzeburg (Scolytinae, Coleoptera). Zookeys.

[131] Bleiker KP, Smith GD, Humble LM (2017). Cold tolerance of mountain pine beetle (Coleoptera: Curculionidae) eggs from the historic and expanded ranges. Environ. Entomol..

[132] Brar GS, Capinera JL, Kendra PE, McLean S, Peña JE (2013). Life cycle, development, and culture of *Xyleborus glabratus* (Coleoptera: Curculionidae: Scolytinae). Florida Entomol..

[133] Jaramillo J, Chabi-Olaye A, Borgemeister C (2010). Temperature-dependent development and emergence pattern of *Hypothenemus hampei* (Coleoptera: Curculionidae: Scolytinae) from coffee berries. J. Econ. Entomol..

[134] Menocal O (2017). Rearing *Xyleborus volvulus* (Coleoptera: Curculionidae) on media containing sawdust from avocado or silkbay, with or without *Raffaelea lauricola* (Ophiostomatales: Ophiostomataceae). Environ. Entomol..

[135] Reid RW, Gates H (1970). Effects of temperature and resin on hatch of eggs of the mountain pine beetle. Can. Entomol..

[136] Rojano F, Ibarra-Juarez LA, Powell J, Salazar R, Lira-Noriega A (2021). Modeling the impact of temperature on the population abundance of the ambrosia beetle *Xyleborus affinis* (Curculionidae: Scolytinae) under rearing conditions. J. Therm. Biol..

[137] Saucedo JR (2017). Nutritional symbionts of a putative vector, *Xyleborus bispinatus*, of the laurel wilt pathogen of avocado, *Raffaelea lauricola*. Symbiosis.

[138] Walgama RS, Zalucki MP (2006). Evaluation of different models to describe egg and pupal development of *Xyleborus fornicatus* Eichh. (Coleoptera: Scolytidae), the shot-hole borer of tea in Sri Lanka. Insect Sci..

[139] Batra LR (1963). Ecology of ambrosia fungi and their dissemination by beetles. Kansas Acad. Sci..

[140] Henriques J, Inácio MDL, Sousa E (2006). Ambrosia fungi in the insect-fungi symbiosis in relation to cork oak decline. Revista Iberoamericana de Micologia.

[141] Biedermann PHW, Klepzig KD, Taborsky M (2009). Fungus cultivation by ambrosia beetles: Behavior and laboratory breeding success in three Xyleborine species. Environ. Entomol..

[142] Cooperband MF (2016). Biology of two members of the *Euwallacea fornicatus* species complex (Coleoptera: Curculionidae: Scolytinae), recently invasive in the U.S.A., reared on an ambrosia beetle artificial diet. Agric. For. Entomol..

[143] Kajimura H, Hijii N (1994). Reproduction and resource utilization of the ambrosia beetle, *Xylosandrus mutilatus*, in field and experimental populations. Entomol. Exp. Appl..

[144] Mizuno T, Kajimura H (2002). Reproduction of the ambrosia beetle, *Xyleborus pfeili* (Ratzeburg) (Col., Scolytidae), on semi-artificial diet. J. Appl. Entomol..

[145] Mizuno T, Kajimura H (2009). Effects of ingredients and structure of semi-artificial diet on the reproduction of an ambrosia beetle, *Xyleborus pfeili* (Ratzeburg) (Coleoptera: Curculionidae: Scolytinae). Appl. Entomol. Zool..

[146] Macedo-Reis LE (2016). Spatio-temporal distribution of bark and ambrosia beetles in a Brazilian tropical dry forest. J. Insect Sci..

[147] Rangel R, Pérez M, Sánchez S, Capello S (2012). Fluctuación poblacional de *Xyleborus ferrugineus* y *X. affinis* (Coleoptera: Curculionidae) en ecosistemas de Tabasco, México. Rev. Biol. Trop..

[148] Peterson AT, Soberón J, Sánchez-Cordero V (1999). Conservatism of ecological niches in evolutionary time. Science.

[149] Etterson JR, Shaw RG (2001). Constraint to adaptive evolution in response to global warming. Science.

[150] Jenkins NL, Hoffmann AA (1999). Limits to the southern border of *Drosophila serrata*: Cold resistance, heritable variation and trade-offs. Evolution.

[151] Guggisberg A (2012). Invasion history of North American Canada thistle, *Cirsium arvense*. J. Biogeogr..

[152] Manrique V (2014). Comparison of two populations of *Pseudophilothrips ichini* (Thysanoptera: Phlaeothripidae) as candidates for biological control of the invasive weed *Schinus terebinthifolia* (Sapindales: Anacardiaceae). Biocontrol Sci. Technol..

[153] Scattolini MC, Confalonieri V, Lira-Noriega A, Pietrokovsky S, Cigliano MM (2018). Diversification mechanisms in the Andean grasshopper genus *Orotettix* (Orthoptera: Acrididae): Ecological niches and evolutionary history. Biol. J. Linn. Soc..

[154] Kostovcik M (2015). The ambrosia symbiosis is specific in some species and promiscuous in others: Evidence from community pyrosequencing. ISME J..

[155] Ibarra-Cerdeña CN, Zaldívar-Riverón A, Peterson AT, Sánchez-Cordero V, Ramsey JM (2014). Phylogeny and niche conservatism in North and Central American triatomine bugs (Hemiptera: Reduviidae: Triatominae), vectors of Chagas’ disease. PLoS Negl. Trop. Dis..

[156] Hadly EA, Spaeth PA, Li C (2009). Niche conservatism above the species level. Proc. Natl. Acad. Sci. USA.

[157] Gilbert, G. S., Magarey, R., Suiter, K. & Webb, C. O. Evolutionary tools for phytosanitary risk analysis: Phylogenetic signal as a predictor of host range of plant pests and pathogens. 10.1111/j.1752-4571.2012.00265.x (2012) 10.1111/j.1752-4571.2012.00265.x PMC3552404 23346231

[158] Formby JP (2018). Cold tolerance and invasive potential of the Redbay ambrosia beetle (*Xyleborus glabratus*) in the eastern United States. Biol. Invasions.

[159] Régnière J, Powell J, Bentz B, Nealis V (2012). Effects of temperature on development, survival and reproduction of insects: Experimental design, data analysis and modeling. J. Insect Physiol..

[160] Pulliam HR (1988). Sources, sinks and population regulation. Am. Nat..

[161] Soberón J, Osorio-Olvera L, Peterson AT (2017). Diferencias conceptuales entre modelación de nichos y modelación de áreas de distribución. Rev. Mex. Biodivers..

[162] Koch FH, Smith WD (2008). Spatio-temporal analysis of *Xyleborus glabratus* (Coleoptera: Circulionidae: Scolytinae) invasion in Eastern U.S. forests. Environ. Entomol..

[163] Seo M, Martini X, Rivera MJ, Stelinski LL (2017). Flight capacities and diurnal flight patterns of the ambrosia beetles, *Xyleborus glabratus* and *Monarthrum mali* (Coleoptera: Curculionidae). Environ. Entomol..

[164] Soberón J, Osorio-Olvera L (2023). A dynamic theory of the area of distribution. J. Biogeogr..

[165] Keitt TH, Lewis MA, Holt RD (2001). Allee effects, invasion pinning, and species’ borders. Am. Nat..

[166] Pironon S (2018). The ‘Hutchinsonian niche’ as an assemblage of demographic niches: Implications for species geographic ranges. Ecography (Cop.).

[167] Gascoigne J, Berec L, Gregory S, Courchamp F (2009). Dangerously few liaisons: A review of mate-finding Allee effects. Popul. Ecol..

[168] Kramer AM, Dennis B, Liebhold AM, Drake JM (2009). The evidence for Allee effects. Popul. Ecol..

[169] Jordal BH, Emerson BC, Hewitt GM (2006). Apparent ‘sympatric’ speciation in ecologically similar herbivorous beetles facilitated by multiple colonizations of an island. Mol. Ecol..

[170] Araújo MB (2019). Standards for distribution models in biodiversity assessments. Sci. Adv..

[171] Gavish Y, O’Connell J, Benton TG (2018). Quantifying and modelling decay in forecast proficiency indicates the limits of transferability in land-cover classification. Methods Ecol. Evol..

[172] Bartos DL, Amman GD (1989). Microclimate: An alternative to tree vigor as a basis for mountain pine beetle infestations. Res. Pap. US Dept. Agric. For. Serv..

[173] Cuddington K, Sobek-Swant S, Crosthwaite JC, Lyons DB, Sinclair BJ (2018). Probability of emerald ash borer impact for Canadian cities and North America: A mechanistic model. Biol. Invasions.

[174] Coeln M, Niu Y, Führer E (1996). Temperature-related development of spruce bark beetles in montane forest formations (Coleoptera: Scolytidae). Entomol. Gen..

[175] Normark BB, Jordal BH, Farrell BD (1999). Origin of a haplodiploid beetle lineage. Proc. R. Soc. B Biol. Sci..

[176] Jordal BH, Normark BB, Farrell BD (2000). Evolutionary haplodiploid Scolytinae radiation of an inbreeding beetle lineage (Curculionidae). Biol. J. Linn. Soc..

[177] Johnson AJ (2018). Phylogenomics clarifies repeated evolutionary origins of inbreeding and fungus farming in bark beetles (Curculionidae, Scolytinae). Mol. Phylogenet. Evol..

[178] Robinson TP (2014). Mapping the global distribution of livestock. PLoS ONE.

[179] Schaub M, Gimenez O, Sierro A, Arlettaz R (2007). Use of integrated modeling to enhance estimates of population dynamics obtained from limited data. Conserv. Biol..

[180] Safranyik L, Whitney HS (1985). Development and survival of axenically reared mountain pine beetles, *Dendroctonus ponderosae* (Coleoptera: Scolytidae), at constant temperatures. Can. Entomol..

[181] McManis AE, Powell JA, Bentz BJ (2019). Developmental parameters of a southern mountain pine beetle (Coleoptera: Curculionidae) population reveal potential source of latitudinal differences in generation time. Can. Entomol..

[182] Elzhov, T. V., Mullen, K. M., Spiess, A. N. & Bolker, B. minpack.lm: R Interface to the Levenberg-Marquardt Nonlinear Least-Squares Algorithm Found in MINPACK, Plus Support for Bounds. R package version 1.2-1. https://CRAN.R-project.org/package=minpack.lm (2016).

[183] R Development Core Team. R: A language and environment for statistical computing. (2020).

[184] Southwood TRE (1978). Ecological Methods.

[185] Barve N (2011). The crucial role of the accessible area in ecological niche modeling and species distribution modeling. Ecol. Modell..

[186] GBIF.org (14 May 2020) GBIF Occurrence Download 10.15468/dl.mcgz2v.

[187] Karger DN (2017). Climatologies at high resolution for the earth’s land surface areas. Sci. Data.

[188] Karger DN (2019). Climatologies at high resolution for the earth’s land surface areas. Sci. Data.

[189] O’Donnell, M. S. & Ignizio, D. A. Bioclimatic predictors for supporting ecological applications in the Conterminous United States. *U.S Geol. Surv. Data Ser.* **691** (2012).

[190] Torres R (2023). Partitioning the effects of habitat loss, hunting and climate change on the endangered Chacoan peccary. Divers. Distrib..

[191] Peterson AT, Martínez-Campos C, Nakazawa Y, Martínez-Meyer E (2005). Time-specific ecological niche modeling predicts spatial dynamics of vector insects and human dengue cases. Trans. R. Soc. Trop. Med. Hyg..

[192] Ochoa-Zavala M (2022). Reduction of genetic variation when far from the niche centroid: Prediction for mangrove species. Front. Conserv. Sci..

[193] Osorio-Olvera L, Yañez-Arenas C, Martíınez-Meyer E, Peterson AT (2020). Relationships between population densities and niche-centroid distances in North American birds. Ecol. Lett..

[194] Osorio-Olvera, L. A. & Hernández, M. A. tenm: Temporal ecological niche models. R package version 1.0. (2022).

[195] Peterson AT, Papeş M, Soberón J (2008). Rethinking receiver operating characteristic analysis applications in ecological niche modeling. Ecol. Modell..

[196] Altamiranda-Saavedra M, Osorio-Olvera L, Yáñez-Arenas C, Marín-Ortiz JC, Parra-Henao G (2020). Geographic abundance patterns explained by niche centrality hypothesis in two Chagas disease vectors in Latin America. PLoS ONE.

[197] Khanal S (2022). Potential impact of climate change on the distribution and conservation status of *Pterocarpus marsupium*, a Near Threatened South Asian medicinal tree species. Ecol. Inform..

[198] Hijmans, R. *raster: Geographic data analysis and modeling. *R package version 3.6–27. https://rspatial.org/raster (2023).

[199] Nathan, R., Klein, E. K., Robledo-Arnuncio, J. J. & Revilla, E. Dispersal kernels: Review (chapter 15). in *Dispersal ecology and evolution* (2012).

[200] Jones KL, Shegelski VA, Marculis NG, Wijerathna AN, Evenden ML (2019). Factors influencing dispersal by flight in bark beetles (Coleoptera: Curculionidae: Scolytinae): From genes to landscapes. Can. J. For. Res..

[201] Calnaido D (1965). The flight and dispersal of shot hole borer of tea (*Xyleborus fornicatus* Eichh., Coleoptera: Scolytidae). Entomol. Exp. Appl..

