# Supplementary material for: Predicting the dispersal and invasion dynamics of ambrosia beetles through demographic reconstruction and process-explicit modeling
Source: Sci Rep. 2024 Mar 30;14:7561. doi: 10.1038/s41598-024-57590-1 (PMC10981740; doi:10.1038/s41598-024-57590-1)
Supplement: Supplementary file 9 — Supplementary Information 2. [file 41598_2024_57590_MOESM9_ESM.docx]

**Predicting ambrosia beetle dispersal and invasion dynamics through demographic reconstruction and process-explicit modeling**

Lucas A. Fadda^1^, Luis Alfredo Osorio-Olvera^2*^, Luis Arturo Ibarra-Juárez^1,2^, Jorge Soberón^4^ & Andrés Lira-Noriega^1,2*^

^1^Instituto de Ecología A. C., Red de Estudios Moleculares Avanzados, Carretera antigua a Coatepec 351, El Haya, C. P. 91073, Xalapa, Veracruz, México

^2^Laboratorio de Ecoinformática de la Biodiversidad, Departamento de Ecología de la Biodiversidad, Instituto de Ecología, Universidad Nacional Autónoma de México, Ciudad de México, México

^3^Biodiversity Institute, University of Kansas, Lawrence, KS 66045, USA

^4^CONAHCyT Research Fellow, Instituto de Ecología A. C., Red de Estudios Moleculares Avanzados, Carretera antigua a Coatepec 351, El Haya, C. P. 91073, Xalapa, Veracruz, México

# **Supplemental files**


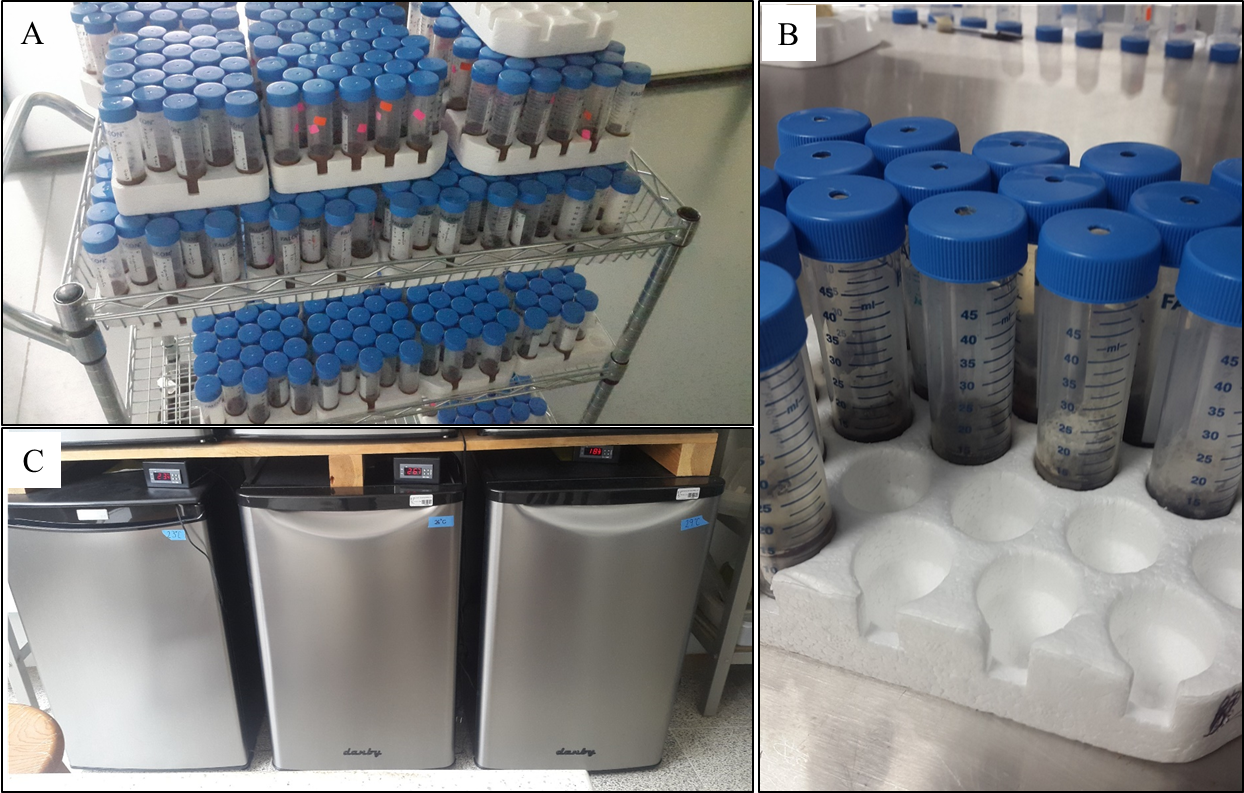


Fig. S1. Laboratory experiment. A) Tubes with culture media prior to beetle inoculation. B) Tubes inoculated with X. bispinatus. C) Chambers used for growth at different temperatures. Photographs taken by Lucas A. Fadda using a 16 MP camera integrated into a Samsung S5 mobile phone.


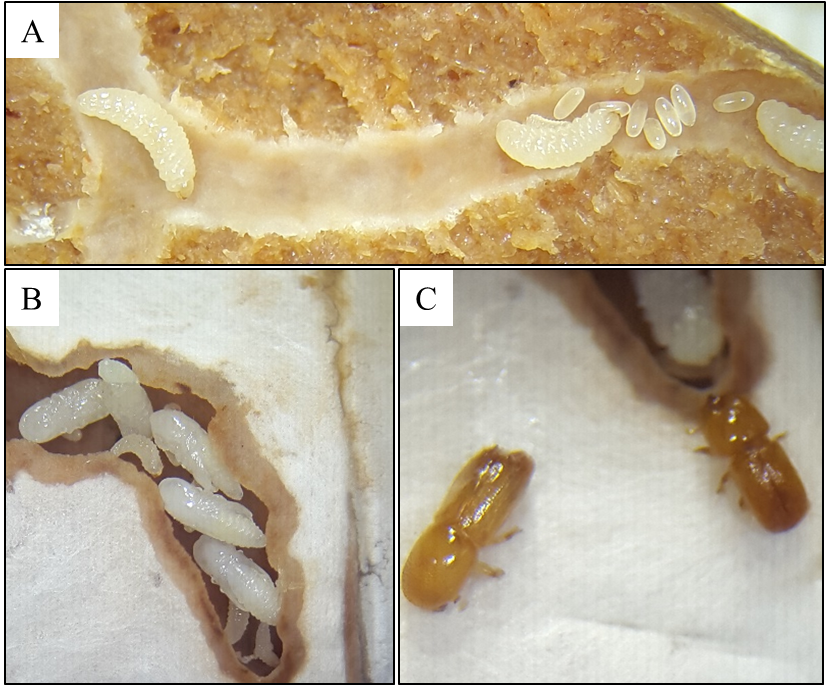


Fig. S2. Individuals of different developmental stages counted in the experiment. A) Eggs and larvae, B) larvae and pupae and C) adult female (left) and male (right). Photographs taken by Lucas A. Fadda using a 16 MP camera integrated into a Samsung S5 mobile phone mounted on a Leica EZ4 stereomicroscope.

Fig. S3. Estimated percent egg hatching of Xyleborus bispinatus inferred from information published by Safranyik & Whitney (1985) for D. ponderosae from a second-order polynomial curve fit.

Table S1. Geographical coordinates used for process-explicit model simulations.

| **Simulation starting points** | | **Site category** | **Latitude** | **Longitude** |
| --- | --- | --- | --- | --- |
| México, Salina Cruz | Port | | 16.175 | -95.194 |
| México, Veracruz | Port | | 19.174 | -96.134 |
| México, Manzanillo | Port | | 19.117 | -104.342 |
| México, Altamira | Port | | 22.392 | -97.939 |
| México, Nogales | Customs | | 31.33236 | -110.96336 |
| Texas, Estados Unidos | Country | | 30.769 | -94.4427 |
| México, Ensenada | Port | | 31.871 | -116.601 |

Table S2. Time in days of the process of dispersal and establishment of the vector-pathogen complex from the simulation start sites all the way to Veracruz, Isthmus of Tehuantepec and the Mexican avocado-growing area (see the considerations mentioned in section 3.3 of the discussion).

| **Simulation** | **Arrival at sites of importance in days** | | | |
| --- | --- | --- | --- | --- |
| **From Gulf of Mexico region** | **Veracruz** | **Isthmus of Tehuantepec** | **Avocado region** | **Time** |
| Puerto de Veracruz | 0 - 155 | 155 – 315 | 315 - 646 | 646 |
| Altamira | 0 - 377 | 377 – 533 | 533 - 760 | 760 |
| Texas | 0 - 1232 | 1232 - 1320 | 1320 - 1700 | 1700 |
| **From Pacific Ocean region** | **Avocado region** | **Isthmus of Tehuantepec** | **Veracruz** | **Time** |
| Salina Cruz | 300-500 | 0-200 | 200-650 | 650 |
| Manzanillo | 0 - 350 | 350 – 700 | 700 - 970 | 970 |
| Nogales | 0 - 1930 | 1930 - 2270 | 2270 - 2670 | 2670 |
| Ensenada | Simulation does not succeed | | | |

Supplementary Videos. Dynamics, abundances, and invasion routes of the ambrosia complex *X. bispinatus*-*H. lauricola* from different sites considered at risk for Mexico. These videos show predictions of potential dispersal dynamics from different starting points seeded at major ports and customs in Mexico. The videos include the following files according to the name of the port (Altamira, Ensenada, Manzanillo, Salina Cruz, Veracruz), customs (Nogales) or locality of co-occurrence of *Xyleborus glabratus* with *X. bispinatus* in Texas. These videos are included in an additional supplementary information set of files.
